# Tissue Response to, and Degradation Rate of, Photocrosslinked Trimethylene Carbonate-Based Elastomers Following Intramuscular Implantation

**DOI:** 10.3390/ma3021156

**Published:** 2010-02-11

**Authors:** Laurianne Timbart, Man Yat Tse, Stephen C. Pang, Brian G. Amsden

**Affiliations:** 1 Department of Chemical Engineering, Dupuis Hall, Queen’s University, Kingston, Ontario K7L 3N6, Canada; E-Mail: laurianne.timbart@chee.queensu.ca (L.T.); 2 Department of Anatomy and Cell Biology, Botterell Hall, Queen's University, Kingston, Ontario K7L 3N6, Canada; E-Mails: myt@queensu.ca (M.Y.T.); pangsc@queensu.ca (S.C.P.)

**Keywords:** *in vivo*, trimethylene carbonate, elastomer, lactide, caprolactone, tissue response, mechanical properties

## Abstract

Cylindrical elastomers were prepared through the UV-initiated crosslinking of terminally acrylated, 8,000 Da star-poly(trimethylene carbonate-co-ε-caprolactone) and star-poly(trimethylene carbonate-co-d,l-lactide). These elastomers were implanted intramuscularly into the hind legs of male Wistar rats to determine the influence of the comonomer on the weight loss, tissue response, and change in mechanical properties of the elastomer. The elastomers exhibited only a mild inflammatory response that subsided after the first week; the response was greater for the stiffer d,l-lactide-containing elastomers. The elastomers exhibited weight loss and sol content changes consistent with a bulk degradation mechanism. The d,l-lactide-containing elastomers displayed a nearly zero-order change in Young’s modulus and stress at break over the 30 week degradation time, while the ε-caprolactone-containing elastomers exhibited little change in modulus or stress at break.

## 1. Introduction 

Thermo- or photoset biodegradable elastomers offer many possible advantages as biomaterials in specific applications. For example, their elasticity and compressibility can be utilized to mimic the mechanical properties of soft tissues such as smooth muscle and cartilage in the preparation of tissue engineering scaffolds suitable for dynamic loading [1,2,3]. Additionally, their elasticity can be utilized to mediate controlled release of peptides and proteins through an osmotic pressure rupturing mechanism [4,5], or can be used in drug delivery applications where a flexible polymer would be appropriate, such as films for the treatment of post-operative adhesions or coil-like geometries in biodegradable stents [6]. 

In particular, photo-initiated crosslinking proceeds rapidly at low temperature, and can provide temporal and spatial control of crosslinking. These characteristics provide a means of fabricating complex structural features [7] and the preparation of protein-loaded devices without denaturing the loaded protein [8,9]. A number of different approaches to preparing photo-crosslinked biodegradable elastomers have been examined, including functionalized prepolymers prepared from poly(glycerol-sebacate) [10], poly(ε-caprolactone-co-lactide) [11,12], and poly(trimethylene carbonate-co-ε-caprolactone) [13,14,15,16]. However, there have been few reports of the degradation rates and mechanisms, changes in mechanical properties, and host tissue response to these materials when implanted *in vivo*. 

Similarly, there have been relatively few reports on the *in vivo* tissue response to and change in properties of trimethylene carbonate-based polymers. Fabre *et al.* examined the nature of the tissue response to subcutaneously implanted linear 23,600 Da, 50 mol % poly(trimethylene carbonate-co-ε-caprolactone) [17]. They reported that this polymer initiated only a mild tissue response with no adverse chronic inflammation. Pego *et al.* found that 232,000 Da, 52 mol % DLLA subcutaneously implanted poly(trimethylene carbonate-co-d,l-lactide) degraded much faster than 89 mol % CL, 154,000 Da poly(trimethylene carbonate-co-ε-caprolactone), and that these polymers initiated a mild tissue response [18]. In a more comparable study, Mizutani and Matsuda examined the tissue response to elastomers prepared from UV-photocrosslinked coumarin-terminated star-poly(trimethylene carbonate-co-ε-caprolactone) [19]. These prepolymers were 5,000 Da, 4-armed star copolymers. Again, the tissue response was reported as mild. None of these studies reported on the degradation rate of the polymers or changes in their mechanical properties during degradation. Moreover, there has yet to be a study wherein the *in vivo* degradation of trimethylene carbonate-based copolymers containing comparable amounts of either d,l-lactide or ε-caprolactone were compared. 

We have recently reported on the *in vivo* degradation of elastomers prepared from UV-initiated crosslinking of 8,000 Da ω,ω´,ω´´-triacrylate [star-poly(trimethylene carbonate)] and ω,ω´,ω´´-triacrylate [star-poly(trimethylene carbonate-co-ε-caprolactone)] [13]. The elastomer prepared from trimethylene carbonate alone degraded through a surface erosion process mediated by the adhesion of activated macrophages and giant cells. After 44 weeks, the elastomer samples had lost about 33% of their initial mass. Elastomers prepared from 8,000 Da prepolymers of trimethylene carbonate copolymerized with equimolar amounts of ε-caprolactone, a hydrolyzable monomer, degraded slowly in a manner consistent with bulk hydrolysis; after 44 weeks these elastomers had lost on average about 21% of their initial mass. These elastomers maintained their Young’s modulus, and stress and strain at break values throughout the degradation period. Our objective in this work was to expand on these findings by exploring the influence of caprolactone composition on the elastomer mechanical properties and *in vivo* degradation rate, and the influence of a more readily hydrolysable monomer, d,l-lactide, on these same properties.

## 2. Results and Discussion

### 2.1. Polymer Properties

The physical properties of the prepolymers are listed in Table 1. The monomer composition and molecular weight of each prepolymer were very close to the theoretical, indicating nearly complete monomer conversion during polymerization. As expected based on their composition, the glass transition temperatures of the prepolymers were very low; the glass transition temperature of linear poly(trimethylene carbonate) has been reported to be −26 °C at a molecular weight of 7,000 Da [21] while the glass transition temperature of poly(ε-caprolactone) is −60 °C [22]. Moreover, the termini of the star copolymers were efficiently acrylated, with degrees of acrylation greater than or equal to 80%. These high degrees of acrylation led to low sol contents after photocrosslinking (less than 5%). 

**Table 1:** Chemical properties of the acrylated star-copolymer prepolymers.

Nature	% TMC	Mn (g/mol)	DA (%)	*T*_g_ (°C)
TMC:CL	67.1	9,300	80.6	−32.5
TMC:DLLA	66.7	8,770	83.8	−13.3

TMC = trimethylene carbonate, CL = ε-caprolactone, DLLA = d,l-lactide, Mn = number average molecular weight, DA = degree of acrylation of the terminal hydroxyl groups, *T*_g_ = glass transition temperature. 

The mechanical properties and glass transition temperatures (*T*_g_) of the photocrosslinked elastomers are listed in Table 2. For comparison, Table 2 also lists values for previously prepared and reported TMC-based elastomers prepared from prepolymers of a similar target molecular weight of 8,000 g/mol [13]. For the CL-containing elastomers, the *T*_g_ decreased as the CL content increased while the Young’s modulus, E, appeared to possess a minimum value at intermediate CL contents. By contrast, all the mechanical properties of the DLLA-containing elastomer were greater in value than the CL-containing elastomer of similar monomer composition. 

**Table 2:** Mechanical properties measured in uniaxial tension of elastomers prepared from TMC-containing copolymer prepolymers.

Comonomer	% TMC	E (MPa)	σ_b_ (MPa)	ε_b_ (mm/mm)	*T*_g_ (°C)
TMC*	100	1.44 ± 0.15	7.23 ± 0.16	11.8 ± 0.5	− 12
CL	67	0.16 ± 0.04	1.43 ± 0.60	10.9 ± 0.78	− 23
CL*	50	0.83 ± 0.14	1.73 ± 0.58	5.2 ± 1.5	− 42
DLLA	66	0.42 ± 0.09	5.2 ± 0.93	14.8 ± 1.2	4

* from Chapanian *et al.* [13].

**Figure 1:**
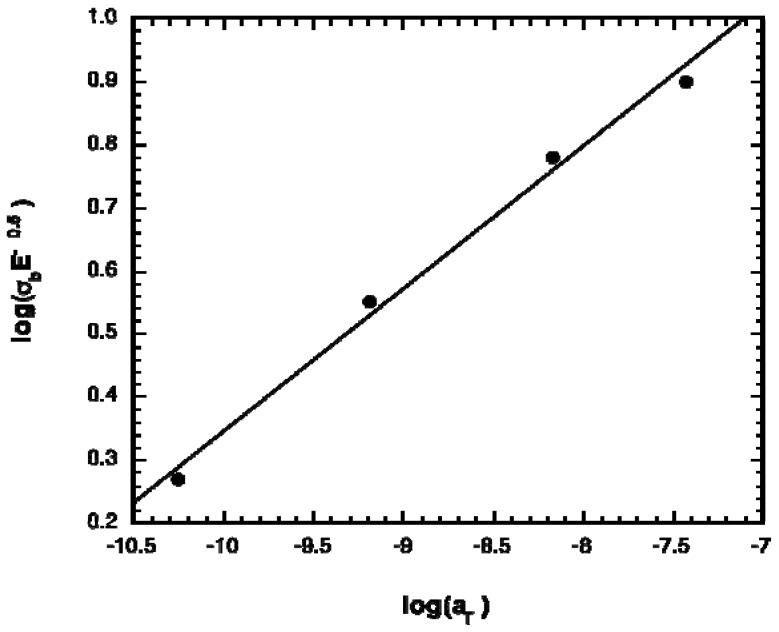
Relationship between shift factor (a_T_) and elastomer strain at break (σ_b_) and modulus (E).

The influence of prepolymer monomer composition on the elastomer mechanical properties can be explained using the theoretical predictions of crack propagation in neo-Hookean materials, as developed previously [20]. In uniaxial tensile testing, failure is considered to occur as a result of crack propagation. For elastomers that deform in a neo-Hookean fashion, the ultimate stress of the elastomer, σ_b_, is given by [24],

(1) $$\sigma_{\text{b}}={\left(\frac{{\text{G}}_{c}\text{E}}{\pi c}\right)}^{\frac{1}{2}}$$

wherein G_c_ is the amount of energy required to advance the crack by unit area and c is the depth of the crack. G_c_ represents the energy dissipated during fracture, and is dependent on the viscoelastic nature of the elastomer. Due to its viscoelastic nature, G_c_ is dependent on the glass transition temperature of the elastomers. This dependence can be described by the Williams-Landel-Ferry (WLF) shift factor, a_T_, using the universal constants [25]

(2) $$\log({\text{a}}_{\text{T}})=\frac{-17.44(\text{T}-{\text{T}}_{\text{g}})}{51.6+(\text{T}-{\text{T}}_{\text{g}})}$$

in which *T*_g_ is the glass transition temperature of the polymer. Figure 1 shows a plot of log(σ_b_E^-1/2^) *versus* log(a_T_), wherein the *T*_g_ of the acrylated prepolymers was used to calculate a_T_. A very good agreement is obtained. This approach provides a means of tuning mechanical properties appropriately, by utilizing the Fox-Flory equation for predicting *T*_g_ along with the universal WLF constants to predict a_T_ and thus the parameter σ_b_(E)^-0.5^. 

### 2.2. Change in Polymer Properties during in Vivo Degradation

**Figure 2:**
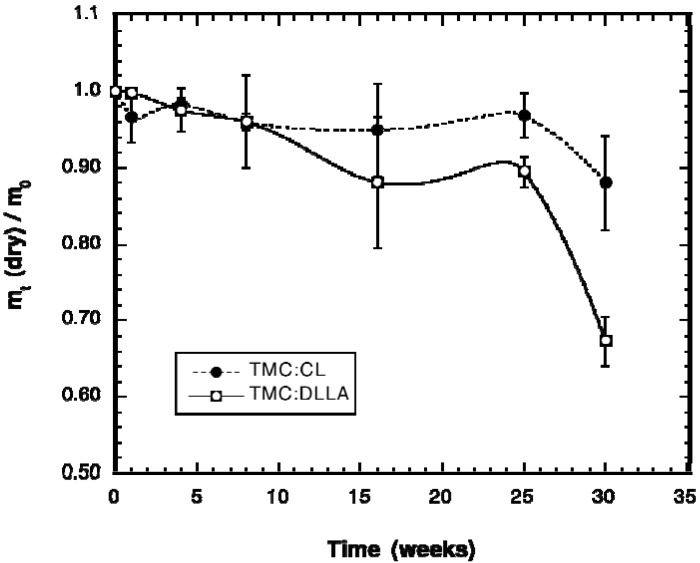
Change in weight during implantation time of elastomers prepared from TMC prepolymers copolymerized with either CL or DLLA. The data is expressed as the ratio of the dry weight of the explanted cylinder (m_t_) to its initial, pre-implant dry weight (m_0_).

The change in weight of the implanted cylinders with time is shown in Figure 2. The data is provided as the ratio of the dry weight of the explanted cylinders to their initial dry weight. Elastomers prepared from the TMC:DLLA prepolymers lost little weight until after week 25; from time 0 to week 25 they had lost only approximately 10% of their initial weight. From week 25 to week 30, a marked increase in mass loss occurred, reaching a total average weight loss of ~32%. By contrast, the TMC:CL elastomers lost even less weight; at week 25 they had lost only ~4–5% weight. They too exhibited an increase in weight loss from week 25 to week 30, but of much less magnitude, reaching only ~12% weight loss at that time. The change in wet weight of the elastomers can be used as an indication of the internal accumulation of degradation products and the change in the crosslink density. 

**Figure 3:**
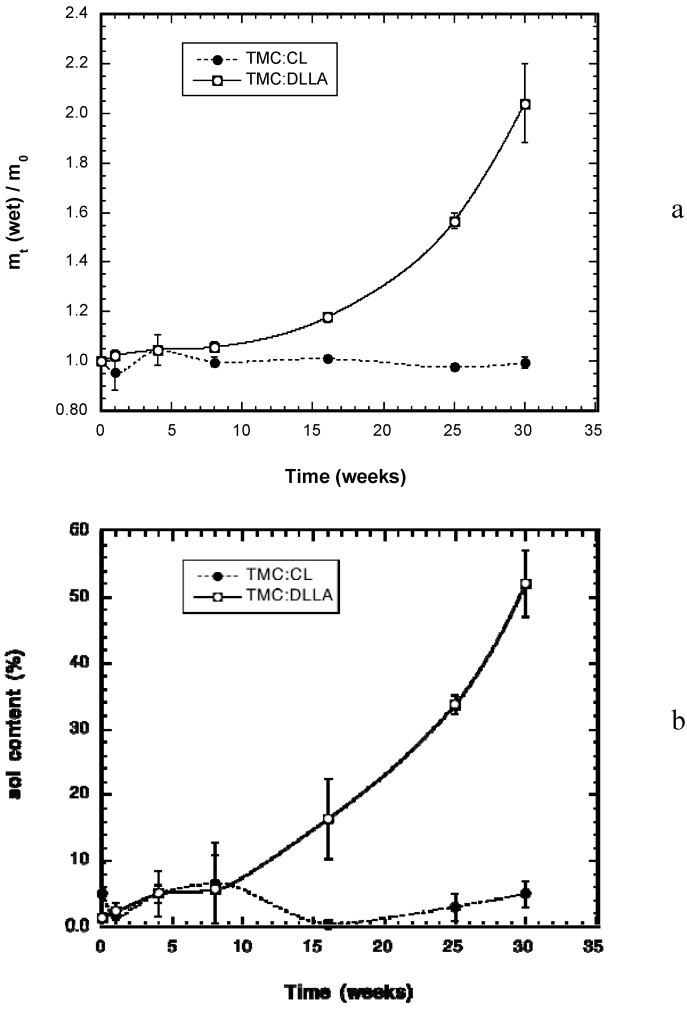
(a) Change in wet weight of the implanted TMC-DLLA and TMC:CL cylinders with time. The data is presented as the ratio of the wet (immediately after explantation) weight (m_t_) to the initial dry weight (m_0_). (b) Change in elastomer sol contents with time during implantation.

The change in wet weight of the explanted elastomer cylinders is given in Figure 3a. The TMC-CL elastomers gained little weight due to absorption of water during the degradation time, while the TMC:DLLA elastomers gained significant amounts of water, more than doubling their initial weight. A marked increase in weight gain was observed following 16 weeks, with the noticeable weight gain accompanying the increase in dry weight loss of the elastomer (Figure 2). Similar to wet weight gain, the sol content of the TMC:DLLA elastomers increased markedly from 16 weeks onward, while that of the TMC:CL elastomers was relatively unaffected (Figure 3b).

**Figure 4:**
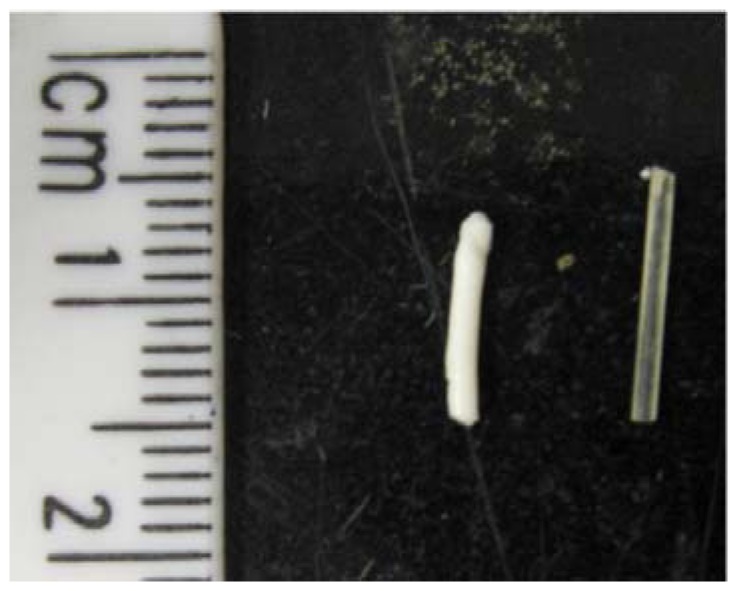
Photograph of the explanted cylinders at week 25. The TMC:DLLA cylinder (left) was white and had changed dimension, while the TMC:CL cylinder (right) had remained translucent and had not changed shape.

The differences in degradation rates of the two types of TMC-based elastomers are apparent in a photograph of the explants at week 30 (Figure 4). The TMC:DLLA elastomer was white and had obviously expanded in diameter while shrinking in length. Conversely, the TMC:CL elastomer remained translucent with no apparent change in dimensions.

Unlike photocrosslinked elastomers composed solely of TMC, which degraded in a surface erosion manner [13], the elastomers prepared from copolymers of TMC and DLLA or CL exhibited degradation behaviour that was consistent with bulk erosion. There was little noticeable mass loss for a prolonged period, followed by an abrupt change in degradation rate, as was noted previously with subcutaneously implanted TMC:CL (50:50) elastomers [13]. Moreover, there appeared to be little degradative contribution of macrophages or foreign body giant cells, as indicated by the histology results. The weight loss change was more abrupt for the TMC:DLLA elastomers, due to the greater hydrophilicity contributed by the DLLA monomer to the network. This increased hydrophilicity was particularly noticeable in enhanced water gain of the TMC:DLLA *versus* the TMC:CL elastomers; the wet weight gain of the TMC:DLLA elastomers was significantly greater than that of the TMC:CL elastomers from week 16 onwards. Initially, weight gain is due to the diffusion of water into the bulk of the material. This rate of water movement into the elastomer will increase as the water plasticizes the polymer and as hydrolysis begins [26]. 

The movement of water into the elastomer is due to an osmotic pressure gradient as a result of the production of low molecular weight water-soluble degradation products that are trapped within the bulk of the elastomer. This water movement occurs to a greater extent due to the increased driving force provided by the osmotic activity gradient. This explanation is supported by the work of Brunner *et al.*, who measured significant osmotic pressure inside degrading PLGA microspheres increased as a result of the presence of degradation products [27,28]. Eventually, this weight gain should become a weight loss as significant amounts of degradation products are leached from the bulk of the material. The TMC:CL elastomers did not swell, indicating their greater hydrophobicity and reduced extent of hydrolysis. The large sol contents of the TMC:DLLA elastomers at weeks 16 and 25 are not matched by high weight loss, despite rather high swelling degrees. This result implies that elastic network chains are being cleaved producing acidic/hydroxyl end groups primarily at these time points, causing both an increase in swelling of the elastomer and high water imbibition due to osmotic pressure effects with little weight loss.

**Figure 5:**
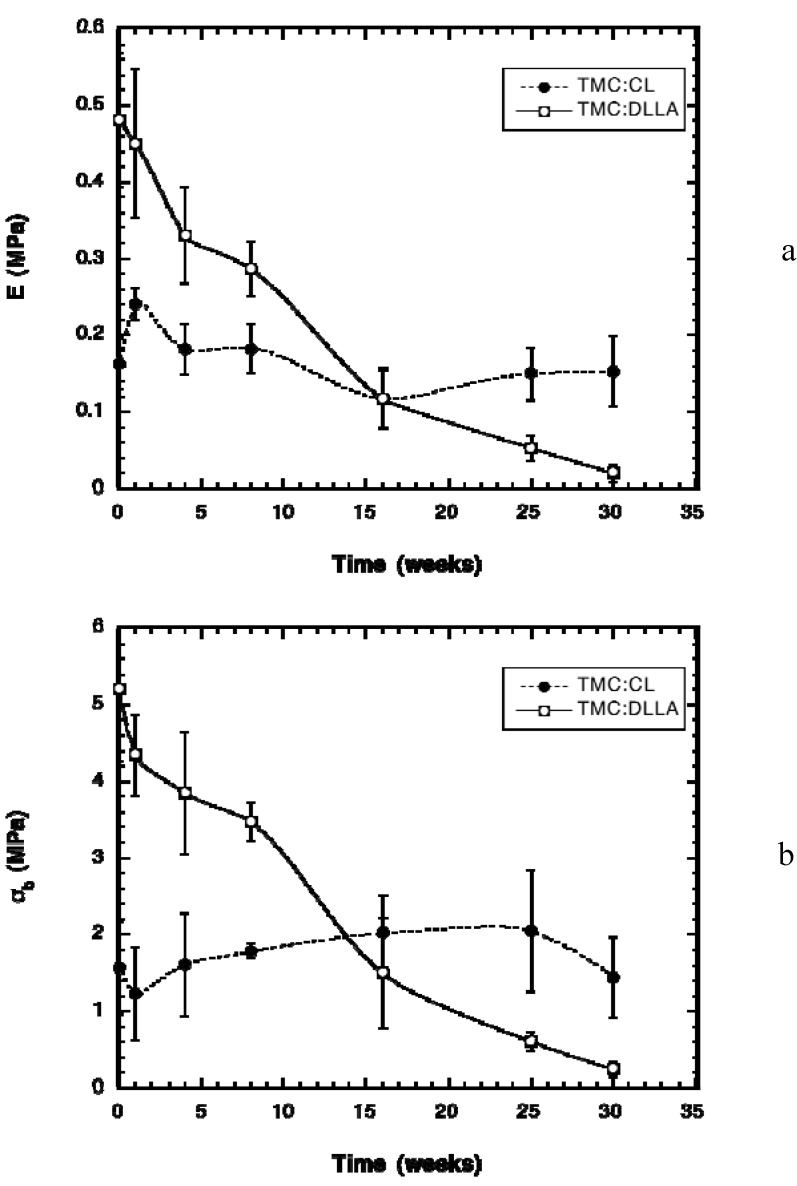
Change in mechanical properties of the elastomers with time, measured in uniaxial tension. (a) Change in Young’s modulus (E). (b) Change in stress at break (σ_b_).

In application, these elastomers need to maintain flexibility, thus changes in their mechanical properties with time were measured. The modulus of the TMC:CL elastomers varied very little during the *in vivo* implantation time (Figure 5a). There was a small increase in modulus at week 1 due to the antiplasticization effect [23], but after this time frame, the modulus returned to very close to the initial value. This result was expected due to the relatively little change in weight (wet and dry) and sol content, which indicated little hydrolysis of the elastomer. The modulus of the TMC:DLLA elastomer decreased nearly linearly up until week 16, after which time the decrease in modulus with degradation time progressed at a slower rate. The stress at break of the elastomers followed the same general trend as the modulus, except without the antiplasticization effect noted for the modulus and the TMC:CL elastomers (Figure 5b). The strain at break of the elastomers did not vary during degradation time.

The manner in which the TMC:DLLA elastomer modulus and stress at break changed with time is in agreement with the picture of elastic network chains being cleaved, leading to a weakening of the material properties with time. This type of decrease in modulus with time is different than that observed with CL:DLLA elastomers, wherein there is an initial lag period of about four weeks during which little change in modulus is noted, and after which a linear decrease in modulus with time occurs [23,29]. The decrease in modulus and stress at break with time of the DLLA elastomers is a result of the greater water absorption of the elastomer due to the increased hydrophilicity of the DLLA monomer. Another contributing factor is the greater propensity of the ester bond in the DLLA monomer to undergo hydrolysis than the ester bond in the CL monomer [30]. This gradual and nearly linear decrease in modulus of the TMC:DLLA elastomer may be suitable for effective tissue engineering scaffold preparation where the load is to be transferred to the developing tissue at a steady rate. 

The strain at break of the elastomers did not vary during the degradation time, which has been noted previously for elastomers prepared from prepolymers of TMC:CL [13] and CL:DLLA [23]. This result is due to the fact that the elastomer will extend to a point determined by the average shortest distance between crosslink points in the network. The average shortest distance between crosslink points is initially only affected by the Mn of the prepolymer and the crosslinking conditions, and would require simultaneous cleavage of multiple elastically effective chains to increase in length. This apparently only happens slowly in these elastomers.

### 2.3. Tissue Response

**Figure 6:**
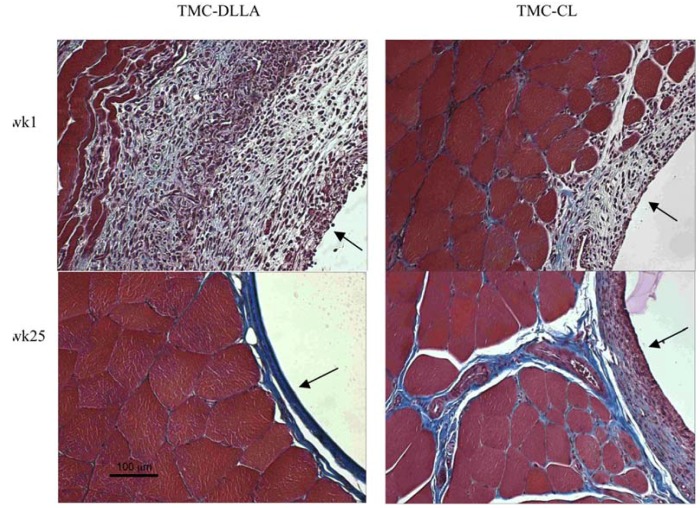
Representative Masson trichrome-stained sections of the tissue surrounding the cylinder implants at week 1 (wk1) and week 25 (wk25). In this staining procedure, collagen stains blue, cytoplasm stains pink/red, muscle stains red, and nuclei stain black. The polymer-tissue interface is indicated by the black arrows.

Over the time frame examined, the implanted elastomers were well tolerated by the host tissue. None of the rats exhibited any weight loss or difficulties in movement. Representative images of the sectioned and stained tissue surrounding the implants are shown in Figure 6 for both the TMC:DLLA and TMC:CL elastomers. By week 1 there was not a clearly demarcated fibrous zone, but there was a recognizable reactive zone of inflammation where there were many inflammatory cells present. The inflammatory response gradually subsided, and by week 25, there was only a relatively thin, fibrous, avascular and relatively acellular and organized reticular tissue layer surrounding the implants. The initial inflammatory response was greater for the DLLA containing elastomers than for the CL-containing elastomers. There was no evidence of the formation of foreign body giant cells at the implant surfaces.

**Figure 7:**
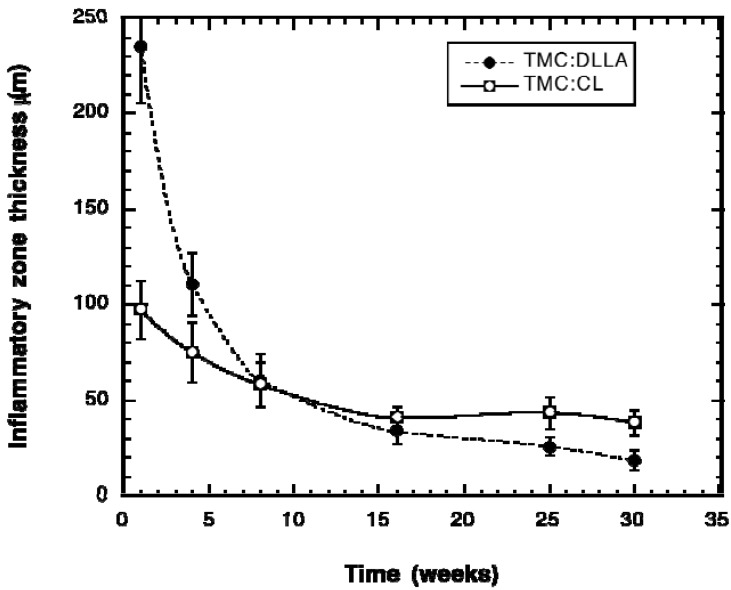
Change in the thickness of the zone of inflammation surrounding the implanted elastomer cylinders with respect to time of implantation.

The change in the thickness of the inflammatory zone surrounding the implants is shown in Figure 7. At weeks 1 and 4, the inflammatory zone is significantly thicker around the TMC:DLLA implants. There was no significant difference between the layer thicknesses from week 8 to week 16, but for weeks 25 and 30, the thickness around the TMC:DLLA elastomer was significantly less than that surrounding the TMC:CL implants.

The initially thicker inflammatory zone around the TMC:DLLA elastomer implants is attributed to their greater stiffness (higher E value), which would have caused a great mechanical irritation to the host tissue. The thickness around the TMC:DLLA elastomer implants decreased with time, in comparison to the TMC:CL elastomer implants wherein the thickness did not decrease appreciably. This result is attributed to the reduction in stiffness and increase in water content with time exhibited by the TMC:DLLA elastomers. Both effects would have made the elastomer softer and less irritating to surrounding tissue.

## 3. Experimental Section

### 3.1. Materials

Unless otherwise stated, all materials were used as received. Glycerol (99.5% purity), stannous 2-ethylhexanoate (95% purity), calcium hydride powder (purity 90–95%), acryloyl chloride (96% purity), triethylamine (99.5% purity), 2,2-dimethoxy-2-phenyl-acetophenone (99% purity) and 4-(dimethylamino)pyridine (99% purity) were used as received from Sigma-Aldrich, Canada. ε-Caprolactone (CL) (99% purity) was purchased from Lancaster Synthesis Inc. and dried and distilled under reduced pressure over calcium hydride. d,l-lactide (DLLA) (99% purity) was used as received from Purac, IL, USA. 1,3-Trimethylene carbonate (TMC) (99% purity) was used as received from Boehringer Ingelheim Corporation, VA, USA. Dimethyl sulfoxide-*d6* (DMSO, 99% purity) was obtained from Cambridge Isotope Laboratories Inc, PQ, Canada.

### 3.2. Synthesis of Star-Copolymers

The star-copolymers were prepared by bulk ring opening polymerization at 140 °C under argon in flame-dried glassware, as previously described [11,13,20]. Glycerol was used as initiator to obtain a three-armed star-copolymer and stannous 2-ethylhexanoate was used as co-initiator/catalyst. The molar ratio TMC:DLLA and TMC:CL was chosen to be 2:1 and the number molecular weight was expected to be 8,000 Da. The reactants were placed at 140 °C for 30 min under 500 mm Hg vacuum in a preheated oven to melt and to remove residual moisture. The ampoule was then purged with argon for 2 min. A solution of catalyst in distilled toluene, at a molar ratio catalyst to initiator equal to 10–3:1 (mol:mol), was added. The solution was stirred and purged again with argon for 2 min, placed in the oven at 140 °C under vacuum for 5 min, and then put under vacuum at room temperature during another 5 min. The ampoule was then flame-sealed under vacuum and placed back at 140 °C under vacuum for 48 hours (72 hours for TMC:CL). The star-copolymers were cooled to room temperature in the sealed ampoule and stored in a desiccator until needed.

### 3.3. Acrylation of the Star-Copolymers

The hydroxyl groups at the chain ends of the star-copolymers were reacted with acryloyl chloride in the presence of the triethylamine and a catalyst, 4-(dimethylamino)pyridine, in anhydrous dichloromethane, as previously described [11,13]. 1.2 mole of acryloyl chloride was used per mole prepolymer terminal hydroxyl. A molar amount of triethylamine equivalent to the number of moles of hydroxyl in the prepolymers was used to scavenge the hydrochloric acid formed during the reaction. Impurities were removed by extracting the polymer with methanol as follows. 80 mL of methanol was added to the dried polymer and the polymer/methanol mixture was stirred one hour at room temperature. The mixture was maintained at −20 °C for 2 hours, then the methanol was decanted. The extraction process was then repeated. The purified product was dried in the fume hood for several days. 

### 3.4. UV Initiated Cross-Linking of the Acrylated Star-Copolymers

Elastomer rods for implantation were formed as follows. The acrylated star-copolymer was dissolved in ethyl acetate at a ratio of 1 g of acrylated product to 1 mL of ethyl acetate. To this solution was added 0.030 g of the UV photoinitiator, 2,2-dimethoxy-2-phenyl-acetophenone (DMPA) per milligram of the acrylated product. The solution was stirred using a vortex mixer and poured into a glass mold cylinder (20 × 1 mm) and covered with a septum. The solution was exposed to UV light at room temperature using a Black-Ray high-intensity long-wave lamp at relative intensity of 100 mW/cm^2^ to yield an elastomer rod. The rod was dried in an oven at 50 °C under vacuum for 1 day. The glass surrounding the rod was then broken to liberate the rod, which was dried for another day at the same conditions. The rods were cut using a scalpel to lengths of 1 ± 0.2 cm. The sol content of the elastomer rods was measured by soaking them in THF, replacing the THF after 1 hour and repeating this process five times. The rods were dried to remove the THF, and the difference in their dry weight before and after soaking in THF used to calculate the sol content.

### 3.5. Polymer Characterization

To confirm the structure and the purity of the star and acrylated star-copolymers and their number average molecular weight (Mn), 1H NMR spectroscopy was performed on a Bruker Avance-500 MHz spectrometer. The samples were prepared in DMSO-*d6*, and run at a concentration of 20 mg/mL at room temperature. The resulting peaks were compared to the solvent peaks relative to tetramethylsilane (TMS) reference. The compositions were determined using the following chemical shifts: TMC (δ = 1.9 ppm, O-CH2-C*H2*-CH2-O), CL (δ = 2.3 ppm, O-C(O)-C*H2*-CH2-), and DLLA (δ = 1.4 ppm, O-C(O)-CH(C*H3*)-). The degree of acrylation and number average molecular weight were calculated using representative end group peaks: TMC and CL (δ = 3.45 ppm, CH2-CH2-C*H2*-OH), DLLA (δ = 1.2 ppm, O-C(O)-CH(C*H3*)-OH , and δ = 6, 6.25, 6.4 ppm for –C*H*=C*H2*).

Thermal characterization of the polymers and rods was achieved using a differential scanning calorimeter TA instruments DSC-Q100. The calorimeter was calibrated with indium and gallium standards prior to use. A heating and cooling rate of 10 °C/min was applied with a temperature program that involved a heating cycle followed by a cooling cycle and a final heating cycle. The sample was heated from −80 °C to 150 °C and held for 1 min at that temperature for the polymers containing CL and from −80 °C to 180 °C for those containing DLLA. This was followed by cooling to −80 °C with a hold time of 5 min at −80 °C. Finally, the sample temperature was increased to 150 or 180 °C. The glass transition temperature (*T*_g_) was taken as the inflection point of the third run endotherm using the internal DSC analysis program. 

The mechanical properties of the rods were measured in uniaxial tension using an Instron tensile tester model 4443. The crosshead speed was set at 500 mm/min according to ASTM D412. All specimens were tested dry at time zero and then immediately after explantation, at room temperature. Data analysis was carried out using Merlin 4.11 Series IX software. 

The sol content of the rods was determined by soaking them 5 times in THF for 1 hour for each soak. Between soaks, the solvent was exchanged with fresh solvent. The rods were then dried in an oven at 50 °C under vacuum for 3 days. The sol content was calculated from the difference in weight before soaking and weight following drying after sol removal in THF.

### 3.6. In Vivo Implantation

*In vivo* biocompatibility and biodegradation of the rods were assessed by intramuscular implantation in 250 g male Wistar rats. The animal experimentation was approved by the Queen’s University Animal Care Committee. Elastomer rods were prepared as described above having average initial diameters of 0.9 ± 0.05 mm, and cut to lengths of 1.2 ± 0.1 mm. The rods, with sol content initially removed, were sterilized in 1 dram vials by UV irradiation for 10 min and soaking in ethanol followed by drying in a sterile flow hood. Three animals were used per time point. The rats were anesthetized prior to implantation of the rods with 2% isoflurane (Baxter Corp.) in oxygen via an Engler ADS 1000 (Benson Medical Industries) at a total flow rate of 0.2 mL/min of O_2_. At a level of surgical anesthesia, (*i.e.*, lack of tail and corneal reflexes), the rats were shaved at the site of implantation. The shaved area was disinfected with hibitane, and 2 cm longitudinal incisions were made. Following implantation, skin incisions were closed with a silk suture. At 1, 4, 8, 16, 25 and 30 weeks following the implantation, the rods were harvested after the rats were anesthetized by an *i.p*. injection of Somnotol (65 mg/kg). The leg was shaved before extracting the rods to check for any inflammatory reaction at the surface of the skin. Two samples from the six, chosen randomly, were retained within the muscle tissue for histology. The remaining rods were explanted, and their mass, diameter and length measured immediately. The mechanical properties and the glass transition temperature were then measured on the wet rods. Following these measurements, the rods were dried under 500 mm Hg vacuum at 50 °C for one week. Their mass was then measured and the percent mass loss calculated. The sol content was also measured as described above. 

The explanted tissues were immediately fixed in 4% paraformaldehyde in phosphate-buffered saline for 16 hours at 20 °C. The tissues were then transferred to 70% ethanol and stored at 4 °C until processing. Processing included dehydration in graded solvents and then embedding in paraffin. Tissue sections were cut at 5µm and stained with Masson’s trichrome. The thickness of the zone of inflammation surrounding the implanted elastomers was calculated as the mean of 8 measurements taken from 5 slides chosen randomly.

### 3.7. Statistics

The data are presented as the mean ± the standard deviation about the mean. Where reported, pair-wise comparisons at each time point were performed using a one-way ANOVA with a Tukey post-hoc analysis. Means were considered significantly different for p < 0.05.

## 4. Conclusions

Elastomers based on UV-photocrosslinked star copolymers of trimethylene carbonate (TMC) copolymerized with either d,l-lactide (DLLA) or Σ-caprolactone (CL) were well tolerated when implanted intramuscularly in rats. These elastomers degraded relatively slowly in a manner consistent with a bulk erosion mechanism. The TMC: DLLA elastomers exhibited a nearly linear loss in modulus with implantation time, a property that may be advantageous for their use as scaffolds for soft, elastic tissue engineering. The modulus change with time of the TMC:CL elastomers, *i.e.,* little to no change over 30 weeks, is more suited for applications where the elastomer is to withstand a load over prolonged periods of time.
